# RabbitKSSD: accelerating genome distance estimation on modern multi-core architectures

**DOI:** 10.1093/bioinformatics/btad695

**Published:** 2023-11-16

**Authors:** Xiaoming Xu, Zekun Yin, Lifeng Yan, Huiguang Yi, Hua Wang, Bertil Schmidt, Weiguo Liu

**Affiliations:** School of Software, Shandong University, Jinan, China; School of Software, Shandong University, Jinan, China; School of Software, Shandong University, Jinan, China; Shenzhen Branch, Guangdong Laboratory for Lingnan Modern Agriculture, Genome Analysis Laboratory of the Ministry of Agriculture, Agricultural Genomics Institute at Shenzhen, Chinese Academy of Agricultural Sciences, Shenzhen, China; School of Software, Shandong University, Jinan, China; Institute for Computer Science, Johannes Gutenberg University, Mainz, Germany; School of Software, Shandong University, Jinan, China

## Abstract

**Summary:**

We propose RabbitKSSD, a high-speed genome distance estimation tool. Specifically, we leverage load-balanced task partitioning, fast I/O, efficient intermediate result accesses, and high-performance data structures to improve overall efficiency. Our performance evaluation demonstrates that RabbitKSSD achieves speedups ranging from 5.7× to 19.8× over Kssd for the time-consuming sketch generation and distance computation on commonly used workstations. In addition, it significantly outperforms Mash, BinDash, and Dashing2. Moreover, RabbitKSSD can efficiently perform all-vs-all distance computation for all RefSeq complete bacterial genomes (455 GB in FASTA format) in just 2 min on a 64-core workstation.

**Availability and implementation:**

RabbitKSSD is available at https://github.com/RabbitBio/RabbitKSSD.

## 1 Introduction

Estimating similarities between genomes serves various purposes, including metagenomic studies (Ondov *et al.* 2019, Elworth *et al.* 2020), phylogenetic analysis (Ondov *et al.* 2016), taxonomic classification and genome clustering (Ondov *et al.* 2016, Yi *et al.* 2021, Xu *et al.* 2023). With the increased data scale of genomic sequences, *k*-mer-based sketching strategies are becoming increasingly important for estimating similarities between genomes.

The *k*-mers are derived from a genomic sequence through a sliding window methodology and transformed into integer hashes. From these hashes, a subset is systematically selected to form what is referred to as a sketch. Notably, the number of chosen hashes, denoted as the sketch size, is substantially smaller in magnitude than the original sequence length. This strategic sketching process enables the expeditious estimation of genomic sequence similarities.

Mash (Ondov *et al.* 2016), BinDash (Zhao 2019), and Dashing2 (Baker and Langmead 2023) are well-known sketching-based applications for estimating genome similarities. Kssd (Yi *et al.* 2021) is another novel sketching technique, which can be used to estimate similarity or containment coefficiency between genomes by *k*-mer substring space decomposition.

Kssd selects a subset of positions by randomly shuffling the total *k*-mer substring space and partitioning it into *N* sub-spaces, where *N* denotes the dimensionality reduction level. One of them is then chosen to compose a sketch. Jaccard and containment similarity of two genomic sequences *A* and *B* are estimated by means of their sketches as $J(A,B)\approx \frac{\left|S(A)\cap S(B)\right|}{\left|S(A)\cup S(B)\right|}$ and $C(A,B)\approx \frac{\left|S(A)\cap S(B)\right|}{\text{min}(|S(A)|,|S(B)|)}$, where S(A) and S(B) denote the sketch of sequence *A* and *B*. The Kssd distances including Mash distance ($D_{m}$) and alignment-and-assembly-free (AAF) distance ($D_{a}$) are computed as $D_{m}(A,B)=-\frac{1}{k}\ln\frac{2J(A,B)}{1+J(A,B)}$ and $D_{a}(A,B)=-\frac{1}{k}\ln C(A,B)$, where *k* is the *k*-mer length. In addition, Kssd uses a one-to-one hash function to avoid the hash collision problem, ensuring the accuracy of set operations on sketches. Subsequently, variants are enriched for population genomics analyses by subtracting the set of *k*-mers associated with a reference dataset from a given dataset.

The mentioned applications provide a series of function modules for genome analysis, such as *sketch* (generate sketch files) and *dist* (compute pairwise distances between reference and query genomes). However, due to sub-optimal implementations, they usually cannot take full advantage of the resources of modern multi-core platforms. This establishes the need of a highly optimized genome similarity estimation tool on commonly used CPUs. Even though Kssd features algorithms with low computational complexity, it exhibits limited robustness and sub-optimal performance. Furthermore, when applied to large-scale genomes, Kssd requires a large amount of compute and storage resources which motivates the need for more efficient software tools.

We propose RabbitKSSD, a highly optimized genome distance estimation tool featuring load-balanced task partitioning, fast I/O, efficient intermediate result accesses, and high-performance data structures. Our evaluation shows that RabbitKSSD gains speedups of at least 5.7× and 16.5× on the most time-consuming kernels of sketch generation and distance computation compared to Kssd on typical workstations. Furthermore, it clearly outperforms Mash, BinDash, and Dashing2.

## 2 Methods

**Figure 1.**
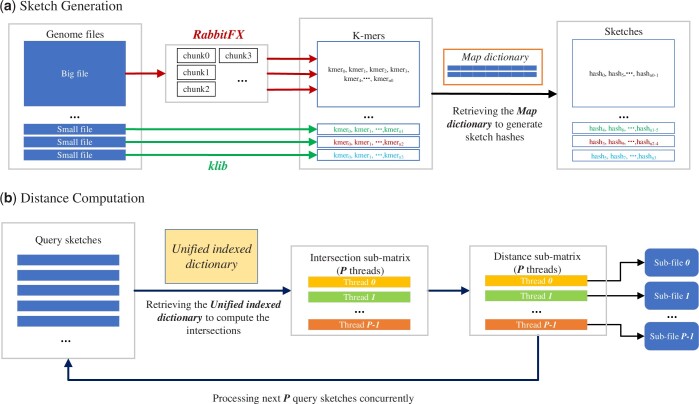
Overview of the RabbitKSSD pipeline: initially, the genome files undergo parsing to extract *k*-mers, which are subsequently used to generate sketches (sketch generation). Following this, the integrated pipeline computes pairwise distances among these genome sketches by retrieving the *unified indexed dictionary* (distance computation)

RabbitKSSD adopts the Kssd algorithm for estimating the similarities between genomes (see Supplementary Section 1.1 for an overview of Kssd). To accelerate time-consuming sketch generation and distance computation, RabbitKSSD relies on a highly tuned task partitioning strategy for load balancing and efficiency. Furthermore, we use a high-performance I/O framework for parsing genome sequences and writing to output files to achieve better thread scalability. We introduce a unified-indexed-dictionary strategy for distance computation to retrieve sketch hashes, which results in enhanced efficiency and robustness. Figure 1 illustrates the RabbitKSSD pipeline, focusing on the time-consuming phases of sketch generation and distance computation. In addition, we propose a producer-consumer multi-threading strategy to accelerate single-threaded sketch set operations (see Supplementary Section 2.3 for details).

### 2.1 Sketch

The first step of sketch generation is to parse the genome sequence files. Kssd uses pipe-streamed I/O APIs to open the genome files in multi-threaded mode, reading one genome file per thread. This may cause load imbalance among multiple threads when the genome files exhibit variations in size. The streamed APIs are also not efficient due to thread-safety issues in multi-threading mode. Furthermore, when the genome number is less than the number of threads, Kssd can often not fully utilize all available CPU cores.

To address these problems, we integrate high-performance sequence parsing tools, including RabbitFX and *klib*. We classify genome input files as ‘big’ and ‘small’ for balanced task partition across multi-threading sketching. RabbitFX is a single-produce-multiple-consumer sequence parsing tool and supports parsing one genome file with multiple threads (Zhang *et al.* 2023). RabbitKSSD parses relatively large genome files with RabbitFX, using multiple threads per file. Subsequently, smaller files are parsed by *klib* using one thread per genome. See Supplementary Section 2.1.1 for details of the partition strategy. The balanced task partition strategy avoids the overhead of waiting for ‘slow’ threads, thereby improving the overall sketching performance. Furthermore, we propose a streaming strategy for sketch generation by shell scripts for thread safety consideration when processing remote data.

When generating sketches, we consider M k-mers and a dimensionality reduction level of *N*. *k*-mers are partitioned into *N* parts, and about $\frac{M}{N}\;k$-mers are chosen for composing sketches. For each *k*-mer, Kssd retrieves the *shuffled dictionary*, created by shuffling the selected hash bits to determine whether this *k*-mer should be added to the sketch. These retrieving operations, however, cause memory access overhead. RabbitKSSD reduces this overhead by using a *map dictionary*, created by the valid hash bits. Our *map dictionary* is implemented by a high-performance robin-hood-hashing map data structure. More details about the *shuffled dictionary* and *map dictionary* are outlined in Supplementary Section 2.1.2.

### 2.2 Dist

Mash distance and AAF distance are based on computing set intersections of sketch pairs. Compared to the frequently used Mash, Kssd is more efficient to compute pairwise intersections (see Supplementary Section 2.2.1 for details). Kssd and RabbitKSSD employ an *indexed dictionary* to store the reference IDs and their associated hash values. Computing the intersections between a query sketch and reference sketches relies on retrieving the query sketch hashes from the *indexed dictionary*. When computing all-vs-all distances between a reference dataset (*m* genomes) and a query dataset (*n* genomes), Kssd maintains an intersection matrix of size of m⋅n. When hash bits exceed 32 (Kssd stores the hashes as 32-bit unsigned integers), Kssd needs to divide the *indexed dictionary* into multiple sub-dictionaries, as shown in Supplementary Section 2.2.2. Given that the combined size of multiple sub-dictionaries often surpasses the available main memory capacity, it needs to update the intersection matrix in multiple passes according to each sub-dictionary. Consequently, Kssd must store the intersection matrix as an intermediate result until the last sub-dictionary is retrieved. The whole intersection matrix may exceed the main memory size when processing large-scale datasets. Kssd thus maps this matrix to hard disk to avoid memory failure.

In RabbitKSSD, we employ 64-bit unsigned integers as hash values when the hash bit surpasses 32. Since the hash value range greatly exceeds the total number of hashes in the sketches, many hash values within this range do not correspond to any entries in the sketches. Storing the indices of all hashes within this range consumes excessive resources and proves to be inefficient. To address this issue, we construct a constrictive *unified indexed dictionary* that exclusively includes hashes present in the sketches. The retrieval of each sketch’s hashes is implemented using a high-performance robin-hood unordered_map. When processing a query sketch, RabbitKSSD retrieves each hash value from the query sketch and identifies all reference IDs associated with these hashes to calculate intersections. Unlike the approach in Kssd, RabbitKSSD utilizes the *unified indexed dictionary* encompassing all reference IDs, thereby eliminating the necessity for intermediate storage of the intersection matrix. As a result, this approach allows for dividing the intersection matrix into individual query lines, obviating the need for the entire matrix to persist on hard disk. These lines are processed concurrently by multiple threads. Consider *P* threads, instead of the complete matrix, RabbitKSSD only needs to hold P⋅m intersection elements in memory, which is much more efficient than accessing the intersection elements on hard disk (see Supplementary Section 2.2.2 for details). In addition, to avoid file writing conflicts and accelerate retrieval speed, RabbitKSSD outputs results using multi-threading to multiple sub-files (see Supplementary Section 2.2.3 for details). Each thread preserves its results within a memory buffer and then writes them to file in buffer-sized units to avoid frequent writing overhead. A global indexed file is used to locate the distances in the sub-files.

## 3 Results

RabbitKSSD is written in C++. It is compared to the latest versions of Mash (v.2.3), BinDash (v.0.2.1), Dashing2 (v.2.1.14), and Kssd (v.2.21). Experiments are conducted on two Linux workstations:

**W1:** dual 32-core Xeon platinum 8375C CPUs, 256 GB DDR4 RAM, Samsung SSD 980 PRO 1TB.

**W2:** dual 24-core AMD EPYC 7402 CPUs, 256 GB DDR4 RAM, Samsung SSD 970 EVO Plus 2TB.

We have measured the performance of these tools using the NCBI RefSeq bacteria genomes, which consist of 455 GB in FASTA format (more details of this dataset are shown in Supplementary Section 3.1). Table 1 shows the runtime and peak memory footprint comparison of these tools on the RefSeq bacteria dataset on W1 and W2 with 64 and 48 threads, respectively. The evaluation of these tools contains generating sketches from origin genome sequences (sketch) and computing all-vs-all distance on sketches (dist). The experiment scripts of evaluating these tools are shown in Supplementary Section 3.5. In a comprehensive evaluation, RabbitKSSD consistently demonstrates superior performance compared to other tools, achieving a performance boost of 2.5–17.5 times while maintaining a modest memory footprint. RabbitKSSD can also achieve comparable or better accuracy with other tools (see Supplementary Section 3.2 for details).

**Table 1.** Performance comparison of Mash, BinDash, Dashing2, Kssd, and RabbitKSSD on the RefSeq bacteria dataset.

Platform	Tools	Run time (s)			Speedup			Peak memory usage (GB)		
		Sketch	Dist	Overall	Sketch	Dist	Overall	Sketch	Dist	Overall
W1	Mash	169.5	1329.5	1499.0	2.3	129.1	17.5	4.0	2.8	4.0
	BinDash	163.9	53.2	217.1	2.2	5.2	2.5	**0.4**	**0.7**	**0.7**
	Dashing2	112.7	523.3	636.0	1.5	50.8	7.4	1.7	45.4	45.4
	Kssd	435.2	204.4	639.6	5.8	19.8	7.5	2.3	2.6	2.6
	RabbitKSSD	**75.2**	**10.3**	**85.5**	–	–	–	1.2	4.2	4.2
W2	Mash	272.7	2025.2	2297.9	2.1	90.8	15.1	3.5	2.6	3.5
	BinDash	355.0	78.6	433.6	2.7	3.5	2.8	**0.4**	**0.7**	**0.7**
	Dashing2	209.7	937.2	1146.9	1.6	42.0	7.5	1.5	37.2	37.2
	Kssd	745.7	367.8	1113.5	5.7	16.5	7.3	1.8	2.4	2.4
	RabbitKSSD	**130.0**	**22.3**	**152.3**	–	–	–	0.9	4.1	4.1

Best runtime and peak memory usage are indicated in bold.

The output of RabbitKSSD is identical to Kssd for all operations. For the sketch operation, RabbitKSSD gains speedups of 2.3× (2.1×), 2.2× (2.7×), 1.5× (1.6×), and 5.8× (5.7×) compared to Mash, BinDash, Dashing2, and Kssd on W1 (W2). For the I/O-intensive sketch operation, RabbitKSSD can reach the peak I/O speed of the fast SSD with multiple threads. For all-vs-all distance computation, using the default sketch size, same *k*-mer size, and the maximum output distance threshold of 0.05, RabbitKSSD achieves speedups of 129.1× (90.8×), 5.2× (3.5×), 50.8× (42.0×), and 19.8× (16.5×) compared to Mash, BinDash, Dashing2, and Kssd on W1 (W2). RabbitKSSD achieves overall speedups of 17.5× (15.1×), 2.5× (2.8×), 7.4× (7.5×), and 7.5× (7.3×) compared to Mash, BinDash, Dashing2, and Kssd on W1 (W2). Apart from Dashing2, the other tools have similar memory footprints for peak memory usage. Instead of mapping the intersection matrix to hard disk in Kssd, RabbitKSSD splits the intersection matrix into lines and preserves these lines in memory. Thus, the memory footprint of RabbitKSSD is slightly higher than Kssd. Moreover, RabbitKSSD exhibits a higher memory footprint compared to BinDash, as it is required to store the *unified indexed dictionary* in memory for efficient retrieval during the computation of pairwise distances.

Furthermore, for the multi-threaded sketch generation and distance computation, RabbitKSSD achieves significantly better thread scalability than Kssd (see Supplementary Section 3.3 for details). For example, RabbitKSSD achieves a parallel efficiency of 65.7% (63.7%) while Kssd only exhibits a parallel efficiency of 30.9% (33.1%) on W1 (W2). For the set operations, RabbitKSSD achieves considerable performance improvement as well, see Supplementary Section 3.4.

In conclusion, RabbitKSSD is a useful and efficient tool for comparing large-scale genome datasets on modern multi-core platforms.

## Supplementary Material

btad695_Supplementary_Data

## Data Availability

The data underlying this article are available in the GenBank Nucleotide Database at https://www.ncbi.nlm.nih.gov/genbank/release/249, NCBI Reference Sequence Database release 211 at ftp://ftp.ncbi.nlm.nih.gov/refseq/release, and 1000 Genomes Project phase 3 at https://www.ncbi.nlm.nih.gov/bioproject/PRJEB31736.
