# Supplementary material for: RabbitKSSD: accelerating genome distance estimation on modern multi-core architectures
Source: Bioinformatics. 2023 Nov 16;39(11):btad695. doi: 10.1093/bioinformatics/btad695 (PMC10681859; doi:10.1093/bioinformatics/btad695)
Supplement: btad695_Supplementary_Data [file btad695_supplementary_data.pdf]

RabbitKSSD supplementary

September 2023

1 Preliminaries

1.1 Kssd algorithm overview

RabbitKSSD employs the Kssd algorithm strategy for estimating distances between genomes. This algorithm involves the generation of sketches from genome sequences and subsequently computing pairwise distances between these sketches.

1.1.1 Sketch generation

Kssd introduces the concept of a "k-mer substring selection pattern (kssp)", represented as a "01" string. Within this pattern, only the positions marked as "1" define the k-mer substring, referred to as the selected substring. This selected substring is randomly shuffled and partitioned into N subspaces, with one of these subspaces chosen to generate the sketch. Consequently, the dimensionality reduction level is defined as N.

Kssd incorporates three parameters for sketch generation: $half_K$, *space*, and *drlevel*. In the process of generating hashes for k-mers, Kssd employs bit encoding, converting each nucleotide into a 2-bit binary value. Specifically, "A", "C", "G", and "T" are encoded as "00", "01", "10", and "11", respectively. The $half_K$ parameter sets the k-mer size, with a default value of 20 ($half_K = 10$), resulting in an initial encoding hash bit count of 40. The *space* parameter controls the space allocated to the selected substring and the *shuffled dictionary*, with a default value of 6, corresponding to 24 space bits. The *drlevel* parameter determines the dimensionality reduction level N ($N = 2^{4 \cdot drlevel}$), with a default *drlevel* of 3, yielding an N of 4096.

As depicted in Figure 1, Kssd generates symmetric kssp encoding hashes based on the $half_K$ and *space* parameters. The *shuffled dictionary* is formed by shuffling the selected codes within the range of 0 to $2^{24} - 1$. Subsequently, the first $\frac{1}{N}$ selected codes are designated as valid codes, and their corresponding indexes are identified as valid indexes. This restricts the valid index range from

0 to $\frac{2^{4 \cdot space}}{2^{4 \cdot drlevel}} - 1$, and the hash bit of the valid index is $4 \cdot (space - drlevel)$. The final hash is composed of the bits of the valid index along with the non-selected kssp hash bits, resulting in a final hash bit count of $4 \cdot (half_K - drlevel)$. All k-mers are generated from the genome sequences using a sliding-window approach, with only those k-mers having selected codes within the valid code range being chosen to constitute the sketch.

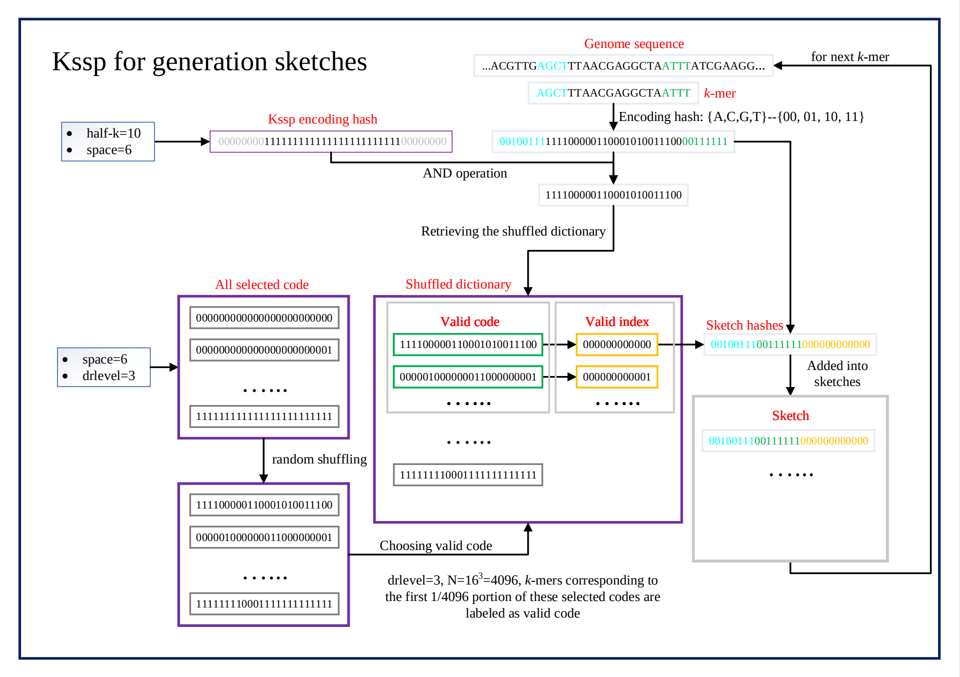

Figure 1: Kssd sketching algorithm.

1.1.2 Distance computation

In Kssd, distance computation relies on the intersection of hash sets within sketches. Kssd generates an *indexed dictionary* for each hash value in the reference sketches to store the IDs of reference sketches containing that specific hash. The process of computing the intersection between a query sketch and the reference sketches involves retrieving each hash value from the query sketch and checking it against the *indexed dictionary*. When the hash bit surpasses 32, Kssd splits the *indexed dictionary* into multiple sub-dictionaries. Consequently, Kssd retrieves the hash values of all query sketches within the sub-dictionaries sequentially. More detailed information is available in Algorithm 2.

Once the computation of the intersection between genomes A and B ($I(A, B)$)

is complete, various similarity metrics can be derived. These metrics include the Jaccard index and the containment coefficient, calculated using the formulas $J(A,B) \approx \frac{I(A,B)}{|S(A)|+|S(B)|-I(A,B)}$ and $C(A,B) \approx \frac{I(A,B)}{min(|S(A)|,|S(B)|)}$. Here, $|S(A)|$ and $|S(B)|$ represent the sketch sizes of genomes A and B. Additionally, the Mash distance and AAF distance (assembly and alignment-free distance) are computed as follows: $D_m(A,B) = -\frac{1}{k}\ln\frac{2J(A,B)}{1+J(A,B)}$ and $D_a(A,B) = -\frac{1}{k}\ln C(A,B)$, where k denotes the k-mer size.

1.2 Parameters

Kssd is characterized by three critical parameters: $half_K$ (the k-mer size), *drlevel* (dimensionality reduction level), and *space* (the space allocated for the *shuffled dictionary*).

1.2.1 K-mer size

Similarities are more sensitive to smaller k values, as smaller k-mers are more likely to find matches. However, there is also a higher chance of random collisions inflating the proportion of shared k-mers, especially when dealing with large genomes using excessively small k-mer sizes. Kssd determines the optimal k-mer size using the equation $k = \log_4 \frac{3g}{2u}$, where g represents the genome size, and u is the specified upper bound for the probability of random k-mer collisions. Both RabbitKSSD and Kssd default to a k-mer size of 20 ($half_K = 10$) to minimize the risk of random collisions, making it suitable for most use cases.

1.2.2 Dimensionality reduction level

The dimensionality reduction level (*drlevel*) is another crucial parameter that directly affects the sketch size. *drlevel* regulates the sub-sampling ratio of k-mers, with only $\frac{1}{16^{drlevel}}$ k-mers being selected to constitute the sketches. In practical terms, this means that the sketch size can be estimated as roughly $L \cdot \frac{1}{16^{drlevel}}$, where L represents the number of k-mers in a genome, approximately equivalent to the genome's length.

Moreover, as illustrated in Figure 1, the number of bits in the final hash values within sketches is calculated as $4 \cdot (half_K - drlevel)$.

1.2.3 *Shuffled dictionary* space

The parameter for controlling the size of the *shuffled dictionary* is denoted as (*space*). The size of the *shuffled dictionary* is determined as $2^{4 \cdot space}$. To improve robustness and efficiency, it's recommended that *space* should be greater than or equal to $drlevel + 2$. The default value for *space* is 6, and generally, there is no need to modify this setting in most use cases.

2 Optimization techniques

2.1 Sketch generation

2.1.1 Balanced task partition

The sketching operation in Kssd encounters two primary performance bottlenecks: parsing genome files and generating sketches through k-mer sampling. Kssd employs pipe-streamed I/O APIs for opening genome files to facilitate sequence parsing. However, when employing multi-threading, these APIs can become inefficient due to thread safety issues. To resolve this issue, RabbitKSSD incorporates two high-performance sequence parsing tools: RabbitFX and *klib*. Additionally, we introduce a task partition strategy aimed at optimizing load balancing.

Kssd adopts a parallel genome file processing strategy, allocating one thread to each genome file. However, this approach can lead to inefficiencies when genome files exhibit substantial variations in size. In such cases, the parsing of the largest file will impede overall performance. It becomes especially problematic when the number of genomes (N_g) is smaller than the number of available threads (P), leading to the underutilization of resources, as only N_g threads are actively engaged in sketch generation, leaving the remaining $P - N_g$ threads idle.

To fully leverage the computational capabilities of modern multi-core workstations, RabbitKSSD categorizes genomes into two distinct types: "big" and "small." This classification is determined based on the thread count (P) and the total genome file size (S_t). Genomes larger than $\frac{S_t}{P}$ are designated as "big" genomes, while the remainder are considered "small" genomes. In scenarios where the total genome count is less than the number of threads, all genomes are treated as "big" genomes.

In Figure 2, we present an exemplary comparison of sketch generation processes between Kssd and RabbitKSSD. When faced with one "big" genome file and three "small" files, Kssd employs four threads to simultaneously read and parse all four files. However, the threads handling the "small" files complete their tasks relatively quickly and subsequently remain idle, awaiting the completion of the thread responsible for processing the "big" file. This distribution of tasks across threads does not achieve an optimal balance for multi-threading. In contrast, RabbitKSSD employs a more efficient approach. It utilizes RabbitFX, a highly efficient sequence parsing tool capable of parsing large genome files using multiple threads simultaneously. RabbitKSSD employs RabbitFX to parse the "big" genome file with multiple threads (e.g. four threads in Figure 2-(b)), including one producer thread and multiple consumer threads. For the "small" files, RabbitKSSD leverages klib and assigns one dedicated thread per file, processing them in descending order of file sizes. This strategic allocation

of resources ensures that RabbitKSSD avoids idle time associated with waiting for the parsing of the "big" file, resulting in a more evenly balanced distribution of tasks and improved overall load balancing.

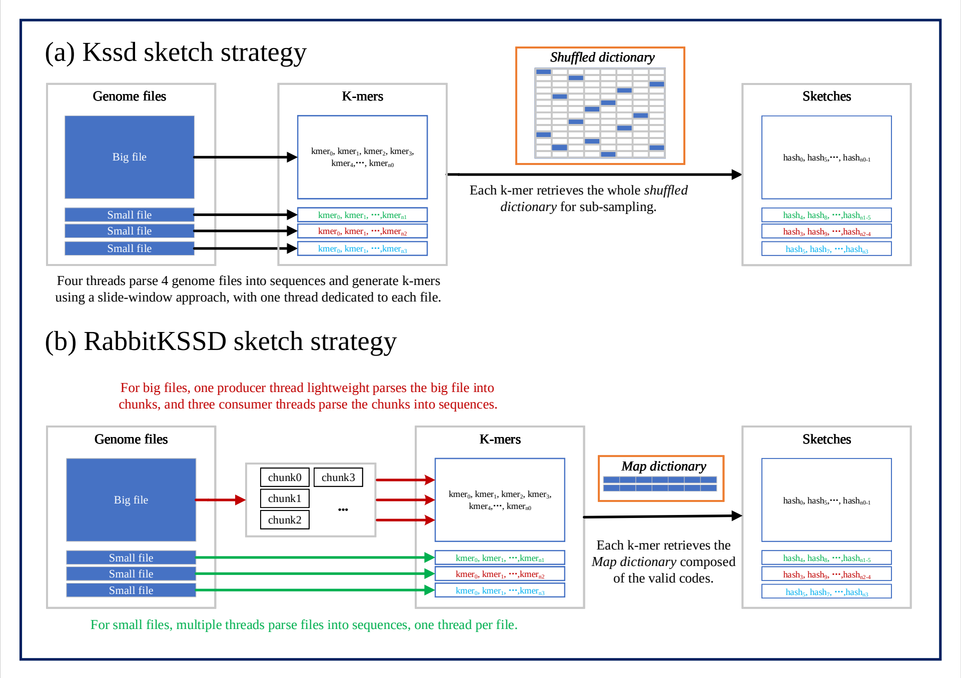

Figure 2: Comparison of sketch generation between Kssd and RabbitKSSD.

2.1.2 Retrieving *map dictionary* instead of the entire *shuffled dictionary*

Kssd generates sketches by sampling k-mers from the entire *shuffled dictionary* of the kssp (k-mer substring selection pattern) space array. These k-mers are generated in a slide-window manner, and the total number of k-mers is approximately to the total genome size. With a dimensionality reduction level parameter (*drlevel*) of l, only a fraction of $1/16^l$ of the k-mers are selected to compose the sketches.

RabbitKSSD constructs a *map dictionary* using a highly efficient robin-hood-hashing map, with a size of $S_{sd}/(16^l)$, considering the size of the entire *shuffled dictionary* in Kssd as S_{sd}. RabbitKSSD then generates sketches by retrieving k-mers from this *map dictionary*, which is only $1/(16^l)$ the size of the whole *shuffled dictionary*. This design results in more efficient sketch generation in RabbitKSSD compared to Kssd.

2.2 Distance computation

2.2.1 Comparison of distance computation between Kssd and Mash

The sketching strategy based on kssp leads to a lower computational complexity compared to Mash, particularly for all-vs-all pairwise distance computations involving large-scale genomes. Algorithms 1 and 2 highlight the distinctions between Kssd and Mash in calculating pairwise distances between reference and query genomes.

For a scenario with M reference genomes and N query genomes, each genome having an average sketch size S, Mash necessitates $M \cdot N$ pairwise intersection computations. Within each pairwise calculation, there are approximately $2 \cdot S$ compare operations. Thus, the overall computational complexity for the Mash strategy is $2 \cdot M \cdot N \cdot S$.

On the other hand, Kssd initially generates an *indexed dictionary* of hash values and genome IDs for reference sketches (depicted by the function *getKssdIndexDict* in Algorithm 2) with a computational complexity of $M \cdot S$. The intersection matrix elements are initialized to 0. For each query sketch and each hash value in the sketch, the intersection matrix is incrementally updated with the genome IDs from the *indexed dictionary.* The complexity of computing the intersection matrix amounts to $N \cdot S \cdot t$, where t represents the average number of genomes containing a specific hash value and $t << M$. Consequently, the total complexity of the Kssd strategy is $(M + Nt) \cdot S$.

When the number of reference genomes (M) and query genomes (N) is large, the condition $(M + Nt) << (2 \cdot M \cdot N)$ holds, indicating that the Kssd strategy has a lower computational complexity than Mash. Additionally, it's worth noting that the operation for each pairwise intersection computation in Mash is also bottlenecked by branch mispredictions.

2.2.2 Comparison of distance computation between RabbitKSSD and Kssd

Though Kssd offers lower computational complexity, it faces practical efficiency challenges, particularly when applied to large-scale genomes. On one hand, it exhibits inefficiency when accessing and updating the intersection matrix stored on the hard disk. On the other hand, it necessitates significant hard disk storage space to accommodate the entire intersection matrix. For instance, when calculating all-vs-all distances for a dataset comprising 1,000,000 genomes, the intersection matrix can consume approximately 4 terabytes of hard disk storage.

The distance computation in both Kssd and RabbitKSSD relies on the intersection of hash sets within sketches. Both tools generate an *indexed dictionary* for each hash, preserving the IDs of reference sketches that contain these hashes. The process of intersecting a query sketch with the reference sketches involves

retrieving the query sketch hashes from the *indexed dictionary*. See details in Algorithms 2 and 3. The crucial aspect of this operation lies in hash value retrieval.

As is shown in Section 1.1, each nucleotide is encoded into 2 bits within these hashes, and the number of hash bits in sketches is determined by the formula $2 \cdot 2 \cdot (half_K - drlevel)$, where $half_K$ and $drlevel$ are parameters reflecting the k-mer size and dimensionality reduction level, respectively. For most scenarios, the count of hash bits is less than 32. However, in cases involving a large k-mer size and a small dimensionality reduction level, the count of hash bits may exceed 32 ($half_K - drlevel > 8$).

In Kssd, the hashes are stored as 32-bit unsigned integers. When the count of hash bits is 32 or fewer, Kssd retrieves the *indexed dictionary* by directly using the hash value as the index. In this case, the index range spans from 0 to $2^{4 \cdot (half_K - drlevel)} - 1$, and the index file has a size of $4 \cdot 2^{4 \cdot (half_K - drlevel)}$ bytes. However, when the count of hash bits exceeds 32, 32-bit integers are insufficient to cover the range of hash values. To address this limitation, Kssd divides the *indexed dictionary* into 2^t distinct sub-dictionaries, where t represents the hash bit value exceeding 32 (see Algorithm 5). Each sub-dictionary has an index file with a size of 16 GB ($4 \cdot 2^{32}$ bytes). For example, when the k-mer size is 26 ($half_K = 13$) and the dimensionality reduction level is 4096 ($drlevel = 3$), the count of hash bit is 40 ($4 \cdot (half_K - drlevel)$). Consequently, Kssd splits the *indexed dictionary* into 256 (2^{40-32}) sub-dictionaries. For each query sketch, Kssd must retrieve hash values from each sub-dictionary. Storing all 256 sub-dictionaries in memory (4 TB in total, with each sub-dictionary having an index file size of 16 GB) becomes impractical for most workstations. Thus, these sub-dictionaries have to be stored in hard disk. Kssd updates the whole intersection matrix by loading each sub-dictionary from the hark disk into RAM. If the intersection matrix is divided into N parts (keeping only a part of the distance matrix in local RAM), these sub-dictionaries must be loaded from hard disk N times, resulting in an unacceptable level of overhead. Thus, Kssd cannot keep only a part of the distance matrix in local RAM, and instead, it stores the whole intersection matrix as an intermediate result to minimize the overhead of loading the sub-dictionaries.

In RabbitKSSD, when the count of hash bits is 32 or lower ($half_K - drlevel \leq 8$), we utilize 32-bit unsigned integers as hash values. The same with Kssd, RabbitKSSD retrieves the *indexed dictionary* by directly using the 32-bit hash value as the index. When the count of hash bits exceeds 32, we employ 64-bit unsigned integers as hash values instead. Thus, the hash value range spans from 0 to $2^{(4 \cdot (half_K - drlevel)}$, and this range greatly exceeds the total number of hashes in the sketches, meaning that many hash values within this range do not correspond to any entries in the sketches. As demonstrated in the previous

example, when the k-mer size is 26, and the dimensionality reduction level is 4096, resulting in a count of hash bits of 40, the hash value range spans from 0 to $2^{40} - 1$. However, the tested RefSeq bacteria dataset contains only about 200 million distinct hashes in the sketches, which is significantly less than the total hash range (2^{40}). As a result, a substantial number of hash values have no associated reference IDs in the Kssd multiple sub-dictionary. Storing the indices of all hashes within total range, including these "empty hashes" (the hashes that never occurred in the sketches), consumes excessive resources and proves to be inefficient. To address this issue, we construct a constrictive *unified indexed dictionary* that exclusively includes hashes present in the sketches. The *unified indexed dictionary* is implemented using a high-performance robinhood unordered_map. The *unified indexed dictionary* eliminates the need to store indices for "empty hashes" and obviates the requirement for multiple sub-dictionaries. When calculating intersections of a query sketch against all references, RabbitKSSD retrieves every hash value from the query sketch and identifies all reference IDs associated with these hashes, see Algorithm 3. Unlike the sub-dictionary-based approach in Kssd, RabbitKSSD utilizes the *unified indexed dictionary* encompassing all reference IDs, thereby eliminating the necessity for intermediate storage of intersection matrix. Consequently, RabbitKSSD can efficiently keep only a part of the distance matrix in local RAM. Consider the parameters of thread count (P), reference genome count (M), and query count (N). RabbitKSSD introduces an efficient strategy that eliminates the need to store the entire intersection matrix on the hard disk, see Figure 3. Instead, it maintains a sub-matrix of size $P \cdot M$ in RAM. This approach significantly enhances both the speed of access and the efficiency of updates. To conduct a comprehensive resource utilization assessment of Kssd and RabbitKSSD, we introduced an additional large-scale dataset denoted as the **Genbank bacteria**. This dataset encompasses 1,009,738 genomes, with a cumulative size of 4.0 terabytes in FASTA format. When performing pairwise distance computations among the genomes within the **Genbank bacteria** using default parameters ($half_K = 10$, $drlevel = 3$), Kssd incurred a computational time of 35,296 seconds (approximately 10 hours) on W1 workstation and 58,020 seconds (about 16 hours) on W2 workstation. Additionally, Kssd necessitated 3.8 terabytes of hard disk space to store intermediate results in the form of an intersection matrix. In contrast, RabbitKSSD completed the same task in significantly shorter times, utilizing 5,516 seconds (roughly 1.5 hours) on W1 workstation and 5,775 seconds (also about 1.5 hours) on W2 workstation. The peak memory footprints of Kssd and RabbitKSSD are 11.2 GB (11.4 GB) and 12.0 GB (12.3 GB) on W1 and W2 workstation, respectively. These results demonstrate that RabbitKSSD achieved notable speed enhancements of 6.4x on W1 workstation and 10.0x on W2 workstation when compared to Kssd. Moreover, RabbitKSSD

effectively mitigates extensive hard disk usage by marginally increasing memory consumption.

We conducted parameter tests on both Kssd and RabbitKSSD, setting $half_K$ to 12 and *drlevel* to 3, using the RefSeq bacteria dataset. During testing, Kssd failed to generate sketches and compute pairwise distances since it was out of memory on both workstations, W1 and W2. In contrast, RabbitKSSD exhibited robust performance. On W1, it took 77.3 seconds for sketch generation and 35.7 seconds for distance computation. On W2, these times were 135.6 seconds for sketch generation and 58.9 seconds for distance computation. Additionally, the peak memory usage for sketch generation and distance computation was 1.4 GB and 3.0 GB on W1, and 1.3 GB and 2.9 GB on W2, respectively. These results demonstrate RabbitKSSD's enhanced robustness compared to Kssd, particularly when the count of hash bits exceeds 32. Notably, RabbitKSSD maintains similar sketching times to cases where hash bits are within the 32-bit limit. Compared to the runtime with default parameters ($half_K = 10$ and $drlevel = 3$) in the main manuscript, the increased computation time in distance computation, for the count of hash bits exceeding 32, can be attributed to the retrieving of *unified indexed dictionary* implemented using an unordered_map. This process is slightly slower than directly accessing the offset array composed by the hash value. However, when count of the hash bit exceeds 32, employing the offset array to generate multiple sub-dictionaries becomes less efficient, similar to Kssd's approach. Furthermore, the use of the *unified indexed dictionary* reduces memory consumption due to a reduction in "empty hashes".

In summary, RabbitKSSD exhibits greater robustness and efficiency across a broader range of parameters compared to Kssd.

2.2.3 Other optimization on distance computation

Furthermore, the outputting of the result file can also pose a performance bottleneck. As illustrated in Figure 3, RabbitKSSD addresses this challenge by merging intersection sub-matrix computation, distance sub-matrix computation, and the generation of distance results into a thread task. Each thread is responsible for generating a sub-file of the final result to prevent writing conflicts. Moreover, each thread conserves its results within a memory buffer and subsequently writes them to the file in buffer-sized units, thereby minimizing frequent writing overhead. Additionally, a global index file is generated to facilitate locating distances within the sub-files. Once the total size of these sub-files falls below 4 GB, they are merged into a single final result file.

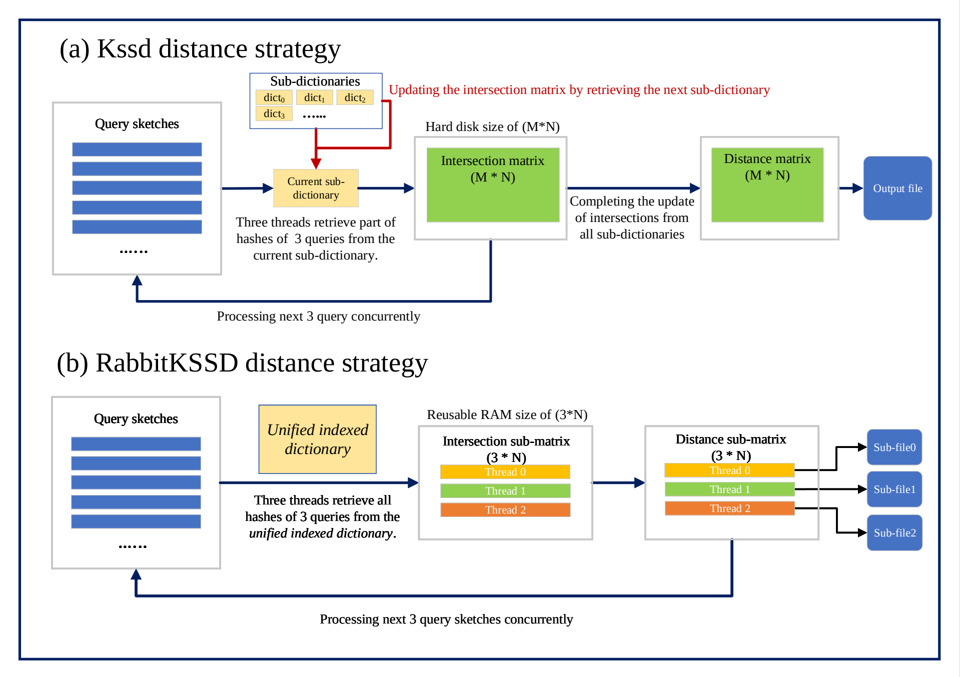

Figure 3: Comparison of distance computation strategy between Kssd and RabbitKSSD.

2.3 Set operations

Kssd and RabbitKSSD use a one-to-one encoding hash function, which differs from the many-to-one hash function used by other tools. This enables the secure subtraction of the sketch hash sets. The reference k-mers will cover the variants between genomes for datasets with the same reference. Therefore, the variants can be enriched by subtracting the k-mer set of the reference from a given dataset. For example, Kssd generated sketches of genomes in the 1000 Genomes Project and then subtracted these sketches by the sketch of the hg38 human reference. The pairwise distances of the remainder sketches were computed for clustering and detecting mislabeled genomes.

The performance of set operations in Kssd is bottlenecked by its single-threaded implementation. To address this limitation, we propose a producer-consumer multithreading strategy to accelerate the set operations. Figure 4 illustrates the comparison between Kssd and RabbitKSSD set subtraction strategies. In the Kssd approach, a single thread is responsible for parsing the sketch file, computing the subtraction, and saving the result to the output file. On the other hand, RabbitKSSD employs a more efficient strategy. It utilizes one

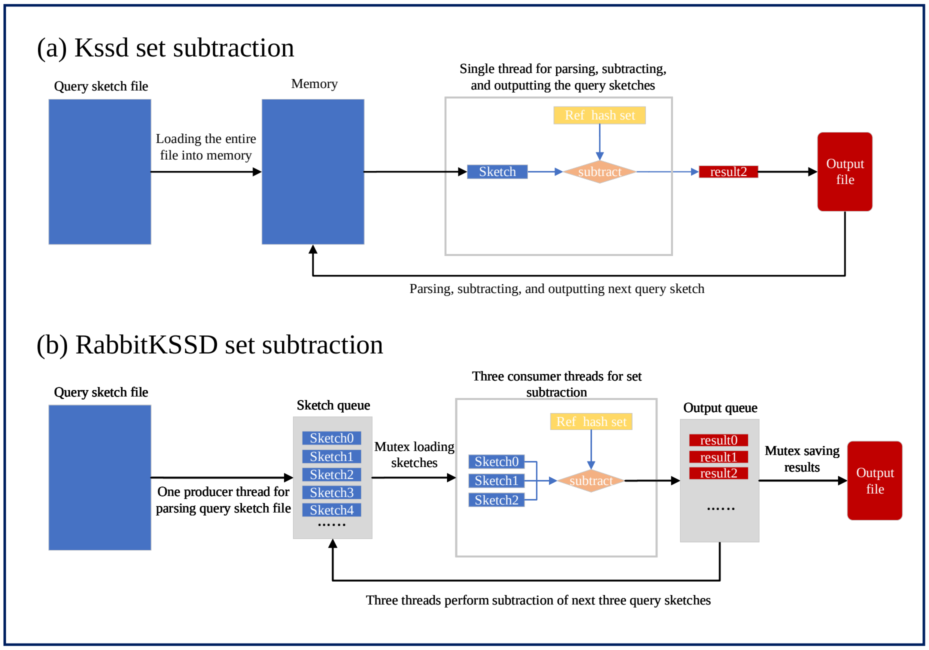

Figure 4: Comparison of set subtraction between Kssd and RabbitKSSD.

thread as a producer to parse the sketch file, while the parsed sketches are placed into a sketch queue. Multiple threads act as consumers, loading sketches from the queue, computing the subtraction, and saving the results to the output file. This parallelized approach in RabbitKSSD allows it to maximize the peak I/O bandwidth of the SSD, resulting in significantly improved set operation performance.

3 Performance evaluation

3.1 Experience data

RefSeq bacteria dataset comprises the complete bacterial genomes from NCBI RefSeq database release 211. This dataset consists of 113,674 genomes, with a cumulative size of 455 GB in FASTA format. The genome sizes within this dataset vary, with the largest being 14,966,964 base pairs, the smallest 143,979 base pairs, and the mean size measuring at 4,287,069 base pairs.

Genbank bacteria dataset comprises the complete bacteriall genomes from NCBI Genbank database release 249. This dataset consists of 1,009,738 genomes,

with a cumulative size of 4.0 TB in FASTA format. The genome sizes within this dataset vary, with the largest being 25,462,055 base pairs, the smallest 176,024 base pairs, and the mean size measuring at 4,374,294 base pairs.

hg38 dataset represents the standard human genome reference and consists of a single genome file, hg_38.fna. This genome is sized at approximately 3.1 GB in FASTA format.

1000Genome dataset corresponds to the NCBI accession PRJEB31736, which involves the sequencing of 2,504 Phase 3 1000 Genome samples at 30X whole genome coverage. In total, 36,940 runs were successfully processed, resulting in a combined data size of approximately 586,862,543,699,180 base pairs, equivalent to around 533.75 terabytes. These runs vary in size, with the largest being 214,967,956,200 base pairs, the smallest being 218,700 base pairs, and the mean size being 15,886,912,390 base pairs. The data retrieval, dumping, and sketch generation processes were performed using a shell script to stream data. At a sketching dimensionality reduction of 4096-fold, the sketches generated from these runs occupy approximately 81 GB in sketch format. After subtracting the sketch of the hg38 human reference, the remaining 36,940 sketches have a combined size of 16 GB.

3.2 Accuracy comparison for estimated Jaccard Coefficients and mutation distance

To assess the estimation accuracy of all the tested tools, we calculate the Sum of Squared Error (SSE) between the estimated Jaccard coefficient and the true Jaccard coefficient. The true Jaccard coefficient between two genomes is determined by comparing the entire sets of k-mers without any subsampling. The RefSeq bacteria database is too large to compute the all-vs-all true Jaccard similarity without subsampling of k-mers. To work with a manageable subset, we use a dataset called the *sub_bact* dataset, consisting of 1000 bacterial genomes, for computing pairwise Jaccard similarity. In this *sub_bact* dataset, the true Jaccard coefficients range from 0.0001 to 1.0, resulting in distances ranging from about 0.0 to 0.3. All the testing tools use default parameters expect for k-mer size. As explained in Section 1.2.1, the optimal k-mer size is about 18, which is large enough to prevent random k-mer collisions. In the experiments, we select k-mer size 16, 18, and 20 to evaluate the similarity between genomes. Table 1 presents SSEs between the estimated Jaccard coefficients and true Jaccard coefficients for these tools on the *sub_bact* dataset. It's worth noting that Kssd and RabbitKSSD yield identical results and, as a result, exhibit the same SSEs. RabbitKSSD and Kssd achieve the lowest SSE when using k-mer sizes of 18 and 20. With a k-mer size of 16, RabbitKSSD and Kssd achieve the second-lowest

SSE.

Table 1: The Sum of Squared Error (SSE) between estimated Jaccard and true Jaccard of Mash, BinDash, Dashing2, Kssd, and RabbitKSSD on the *sub_bact* dataset. Bold indicates the lowest error.

k-mer size	Mash	binDash	Dashing2	Kssd	RabbitKSSD
16	0.7729	**0.5149**	0.5488	0.5340	0.5340
18	0.1740	0.0822	0.1373	**0.0720**	**0.0720**
20	0.0644	0.0369	0.0661	**0.0355**	**0.0355**

Table 2: The Sum of Squared Error (SSE) between estimated Jaccard and true Jaccard of Mash, BinDash, Dashing2, Kssd, and RabbitKSSD on the simulate dataset. Bold indicates the lowest error.

k-mer size	Mash	binDash	Dashing2	Kssd	RabbitKSSD
16	0.0716	0.0346	0.0698	**0.0190**	**0.0190**
18	0.0631	0.0309	0.0585	**0.0168**	**0.0168**
20	0.0542	0.0226	0.0542	**0.0138**	**0.0138**

Table 3: The Pearson correlation coefficients between estimated Mash distance and true mutation distance of Mash, BinDash, Dashing2, Kssd, and RabbitKSSD on the simulate dataset. Bold indicates the highest correlation.

k-mer size	Mash	binDash	Dashing2	Kssd	RabbitKSSD
16	0.6998	0.8921	0.7175	**0.9927**	**0.9927**
18	0.8162	0.9827	0.7409	**0.9939**	**0.9939**
20	0.9879	0.9942	0.9865	**0.9972**	**0.9972**

Kssd utilizes the Pearson correlation coefficient to assess the accuracy of the mutation distance. However, since the ground truth of mutation distances on the real-world RefSeq bacteria dataset is not available, we simulated 900 genome sequences with mutation rates (mutation distances) ranging from 0.1% (0.001) to 30% (0.300), based on a 10,000,000 bp seed nucleotide genome. These mutation rates involve an equal probability of insertion, deletion, and substitution. The true Jaccard coefficients between the seed genome and these mutated genomes vary from 0.005 (mutation distance 0.299) to 0.970 (mutation distance 0.001). Table 2 and Table 3 display the SSEs and Pearson correlation coefficients of these tools on the simulated dataset, respectively. The testing results show that Kssd and RabbitKSSD tend to be slightly more accurate and exhibits better performance than other tools.

In summary, RabbitKSSD achieves at least comparable accuracy to other tools while exhibiting superior runtime efficiency.

3.3 Thread scalability

Figure 5 and Figure 6 depict the thread scalability of RabbitKSSD and Kssd on the 64-core Intel (W1) and 48-core AMD (W2) workstations. RabbitKSSD demonstrates superior thread scalability compared to Kssd for both compute-intensive sketch generation and distance computation operations. Table 4 presents the parallel efficiency of Kssd and RabbitKSSD on the two workstations.

Table 4: The parallel efficiency of Kssd and RabbitKSSD on the 64-core Intel (W1) and 48-core AMD (W2) workstations.

	operation	Kssd	RabbitKSSD
W1	sketch	42.1%	66.0%
	dist	4.9%	63.3%
	sketch&dist	30.9%	65.7%
W2	sketch	44.0%	63.2%
	dist	6.5%	50.7%
	sketch&dist	33.1%	61.4%

For sketch generation, RabbitKSSD achieves near-linear speedup when using up to 48 (32) threads on the Intel (AMD) workstation. The SSD on the Intel (AMD) workstation is a Samsung 980 PRO 1TB SSD (Samsung 970 EVO Plus 2TB SSD) with a peak sequential read speed of 7,000 (3,500) MB/s. RabbitKSSD can process FASTA genome files of up to 6 GB (3 GB) per second with 48 (32) or more threads on the Intel (AMD) workstation. This allows RabbitKSSD to scale effectively up to the peak bandwidth of the SSD.

For distance computation, thanks to the task partition method and efficient output strategy, RabbitKSSD achieves a nearly linear speedup as the number of threads increases. For the task of distance computation conducted on the AMD workstation, the time required to compute all-vs-all distances for the RefSeq bacteria dataset stands at 22.3 seconds (22.2 seconds) when employing 44 (48) threads. In the case of the RefSeq bacteria dataset, consisting of 113,674 genomes, the incremental gain in speed achieved by increasing the thread count does not sufficiently offset the additional overhead introduced by multi-threading.

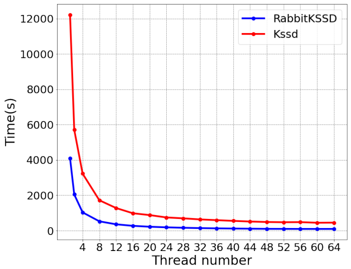

(a) Runtime of sketch generation of RabbitKSSD and Kssd with different numbers of threads

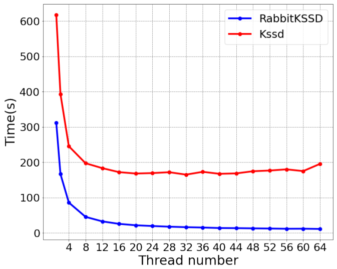

(b) Runtime of distance computation of RabbitKSSD and Kssd with different numbers of threads

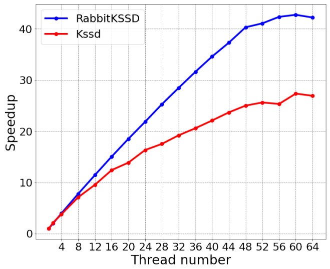

(c) Thread scalability for sketch generation with different numbers of threads

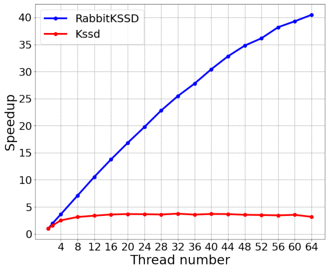

(d) Thread scalability for distance computation with different numbers of threads

Figure 5: Thread scalability of sketch generation and distance computation of RabbitKSSD and Kssd on the RefSeq bacteria dataset on a 64-core Intel workstation.

3.4 Comparative evaluation of set operations between Kssd and RabbitKSSD

We evaluate the performance of set subtraction using the 1000 Genome sketch with Kssd and RabbitKSSD. The 1000 Genome sketch is generated in a streaming fashion by the shell script, with its performance mainly bottlenecked by download and dumping speeds. The resulting 1000 Genome sketch file is 81 GB in size, which is used to generate a 16 GB remainder sketch file by subtracting the hg38 reference sketch. The comparison of sketch subtraction and all-vs-all distance computation of the remainder sketches between Kssd and RabbitKSSD is summarized in Table 5.

As shown in Table 5, RabbitKSSD achieves notable speedups, with performance gains of 7.6x and 14.0x on the Intel (W1) and AMD (W2) workstations, respectively, in the set subtraction phase. This improvement is due to the producer-consumer module in RabbitKSSD, which efficiently processes the

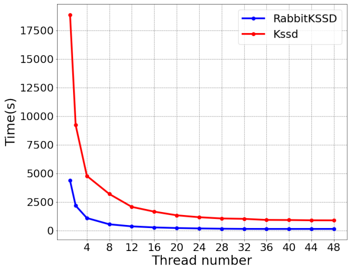

(a) Runtime of sketch generation of RabbitKSSD and Kssd with different numbers of threads

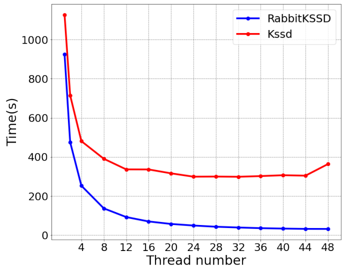

(b) Runtime of distance computation of RabbitKSSD and Kssd with different numbers of threads

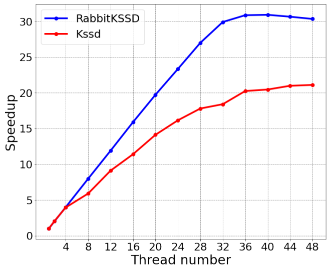

(c) Thread scalability for sketch generation with different numbers of threads

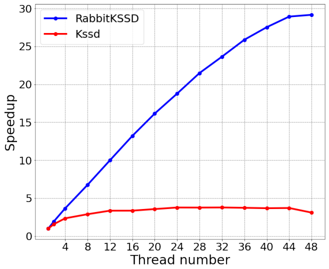

(d) Thread scalability for distance computation with different numbers of threads

Figure 6: Thread scalability of sketch generation and distance computation of RabbitKSSD and Kssd on the RefSeq bacteria dataset on a 48-core AMD workstation.

original sketch files chunk by chunk. In contrast, Kssd's single-threaded module loads the entire original sketch files (81 GB) into RAM, resulting in high memory usage.

It's worth noting that the speedup in the all-vs-all distance computation for these remainder sketches is not as significant as that observed in the RefSeq bacteria dataset (i.e. $\approx$ 3x vs. $\approx$ 16x). The mean sketch size of the 16 GB remainder sketches and RefSeq bacteria sketches is approximately 116,000 and 1,000, respectively. Given the larger number of hash values in the remainder sketches, the most time-consuming aspect of distance computation is retrieving these hash values from the *indexed dictionary* in both RabbitKSSD and Kssd. The performance improvement achieved by RabbitKSSD is not as significant as with RefSeq bacteria dataset due to the most time-consuming *indexed dictionary* retrieving. Nonetheless, RabbitKSSD's robustness and performance still outshine Kssd.

Table 5: Comparison of set subtraction (sub) and all-vs-all distance computation (dist) between RabbitKSSD(RKSSD) and Kssd on 1000Genome sketches.

		Runtime(s)			Peak Memory(GB)			Speedup		
		sub	dist	overall	sub	dist	overall	sub	dist	overall
W1	RKSSD	34.5	374.9	409.4	1.2	34.0	34.0	-	-	-
	Kssd	263.9	953.6	1217.5	80.5	37.2	80.5	7.6	2.5	3.0
W2	RKSSD	36.0	517.7	553.7	1.7	33.9	33.9	-	-	-
	Kssd	504.7	1307.3	1812.0	80.9	37.4	80.9	14.0	2.5	3.3

3.5 Experiment scripts

Build from source:

```
git clone --recursive https://github.com/RabbitBio/RabbitKSSD.git
cd RabbitKSSD
# Compile automatically
./install.sh
# Generate the shuffle files
./init_shuffle.sh
```

Generate sketch (on W1 workstation with 64 threads)

```
# Input is bacteria RefSeq genome directory for Kssd, and bacteria RefSeq genome list for other tools.
# Output is bacteria_sketch directory for Kssd, and bacteria.sketch for other tools.
./mash sketch -l bact.list -o bact.sketch -p 64
./bindash sketch --listfname=bact.list --nthreads=64 --outfname=bact.sketch
./dashing2 sketch -F bact.list -p 64 -o bact.sketch
./kssd dist -L shuf_file/L3K10.shuf -r bact/ -o bact_sketch
./rabbit_kssd sketch -L shuf_file/L3K10.shuf -i bact.list -o bact.sketch
```

Compute all-vs-all distances (on W1 workstation with 64 threads)

```
# Input is bacteria sketch directory of Kssd, and bacteria sketch file for other tools.
# Output is the bacteria.dist for all tools.
./mash triangle -d 0.05 -p 64 bact.sketch.msh >bact.dist
./bindash dist --nthreads=64 --mthres=0.05 --outfname=bact.dist bact.sketch
./dashing2 cmp --cmpout bact.dist --presketched bact.sketch -p 64
./kssd dist -r bact_sketch -o bact_dist -D 0.05 bact_sketch
./rabbit_kssd alldist -i bact.sketch -D 0.05 -o bact.alldist
```

Set union and subtraction

```
# Compute the set union of hg38 sketch
./kssd set -u -o hg38_union hg38_sketch
./rabbit_kssd union -i hg38.sketch -o hg38_union.sketch

# Compute the set subtraction between 1000Genome sketch and hg38 sketch
./kssd set -s hg38_union -o 1000Genomes_sub_sketch 1000Genomes_sketch
./rabbit_kssd sub --rs hg38_union.sketch --qs 1000Genomes.sketch -o 1000Genomes_sub.sketch
```

Algorithm 1 Distance computation strategy of Mash

Input: Sketches array $S_a[M]$ consists of M sketches, sketch array $S_b[N]$ consists N sketches; Each sketch ($S_a[i]$ or $S_b[j]$) is a sorted hash array;

Output: intersection matrix $Common[M][N]$;

```
i ← 0, j ← 0;
for i = 0; i < M; i++ do
    for j = 0; j < N; j++ do
        Common[i][j] ← INTERSECTION(S_a[i], S_b[j]);
    end for
end for

function INTERSECTION(ArrayA, ArrayB)
    common ← 0;
    i ← 0, j ← 0;
    sizeA ← ArrayA.size(), sizeB ← ArrayB.size();
    while i < sizeA and j < sizeB do
        if ArrayA[i] == ArrayB[j] then
            common++;
            i++;
            j++;
        else if ArrayA[i] < ArrayB[j] then
            i++;
        else
            j++;
        end if
    end while
    return common;
end function
```

Algorithm 2 Distance computation strategy of Kssd

Input: Reference sketches array $S_a[M]$ contains M sketches, query sketch array $S_b[N]$ consists of N sketches; Each sketch ($S_a[i]$ or $S_b[j]$) is a hash array; Sketch parameters $half_K$ and $drlevel$.

Output: Intersection matrix $Common[N][M]$;

Variables: $IDFile[]$ is an array of files used for saving the reference IDs ($IDArr[]$) of each hash; $offsetFile[]$ is an array of files used for saving the offset ($offsetArr[]$) of the hashes to retrieve the reference IDs.

function KssdIntersection($S_a[M], S_b[N], half_K, drlevel$)
 for $i = 0; i < N; i++$ **do**
  for $j = 0; j < M; j++$ **do**
   $Common[i][j] \leftarrow 0$;
  end for
 end for
 getKssdIndexDict($S_a[M], half_K, drlevel, offsetFile[], IDFile[]$);

 if $half_K - drlevel > 8$ **then**
  $n \leftarrow 2^{32}$;
  $numDict \leftarrow 2^{4\times(half_K - drlevel - 8)}$;
  for $index = 0; index < numDict; index++$ **do**
   $offsetArr[n] \leftarrow ReadFromFile(offsetFile[index])$;
   $IDArr[] \leftarrow ReadFromFile(IDFile[index])$;
   $updateIntersectionMatrix(S_b[N], index, offsetArr[n], IDArr[], Common[N][M])$;
  end for
 else
  $n \leftarrow 2^{4\times(half_K - drlevel)}$;
  $offsetArr[n] \leftarrow ReadFromFile(OffsetFile[0])$;
  $IDArr[] \leftarrow ReadFromFile(IDFile[0])$;
  $updateIntersectionMatrix(S_b[N], 0, offsetArr[n], IDArr[], Common[N][M])$;
 end if
 return $Common[N][M]$;
end function

Algorithm 3 Distance computation strategy of RabbitKSSD

Input: Sketches array $S_a[M]$ contains M sketches, sketch array $S_b[N]$ consists N sketches; Each sketch ($S_a[i]$ or $S_b[j]$) is a hash array; sketch parameters $half_K$ and $drlevel$.

Output: intersection matrix $Common[M][N]$.

Variables: $IDFile$ is a file used for saving the reference IDs ($IDArr[]$) of each hash. $offsetFile$ is a file used for saving the offset ($offsetArr[]$) of the hashes to retrieve the reference IDs.

```
function RabbitKSSDIntersection(S_a[M], S_b[N], half_K, drlevel)
    getRabbitKSSDIndexDict(S_a[M], half_K, drlevel, offsetFile, IDFile);

    if half_K − drlevel > 8 then
        dictMap ← ReadFromFile(offsetFile, IDFile);
    else
        offsetArr[] ← ReadFromFile(offsetFile);
        IDArr[] ← ReadFromFile(IDFile);
    end if
    for i = 0; i < N; i + + do
        S ← S_b[i].size;
        for j = 0; j < S; j + + do
            hash ← S_b[i][j];
            if half_K − drlevel > 8 then
                start ← 0, end ← dictMap[hash].size;
                for k = start; k < end; k + + do
                    refID ← dictMap[hash][k];
                    Common[j][refID] + +;
                end for
            else
                if hash == 0 then
                    start ← 0, end ← offsetArr[hash];
                else
                    start ← offsetArr[hash − 1], end ← offsetArr[hash];
                end if
                for k = start; k < end; k + + do
                    refID ← IDArr[k];
                    Common[j][refID] + +;
                end for
            end if
        end for
    end for
    return Common[N][M];
end function
```

Algorithm 4 Updating the intersection matrix of Kssd

Input: Query sketch array $S_b[N]$ contains N sketches; Each sketch ($S_b[i]$) is a hash array; *index* is the index of the *indexed dictionary* or sub-dictionary; $IDArr[]$ is the array of reference ID for each hash; $offsetArr[]$ is the array of the offset for retrieving the reference IDs.

Output: Intersection matrix $Common[N][M]$;

```
function updateIntersectionMatrix(S_b[N], index, offsetArr[], IDArr[], Common[][])
    n ← 2^32;
    for i = 0; i < N; i++ do
        S ← S_b[i].size;
        for j = 0; j < S; j++ do
            key ← S_b[i][j] % n;
            curIndex ← S_b[i][j] / n;
            if curIndex == index then
                if key == 0 then
                    start ← 0, end ← offsetArr[key];
                else
                    start ← offsetArr[key − 1], end ← offsetArr[key];
                end if
                for k = start; k < end; k++ do
                    refID ← IDArr[k];
                    Common[i][refID]++;
                end for
            end if
        end for
    end for
end function
```

Algorithm 5 Generating the *indexed dictionary* of Kssd

Input: Reference sketch array $S_a[M]$ contains M sketches; Each sketch ($S_a[i]$) is a hash array; $half_K$ and $drlevel$ are the parameters of k-mer size and dimensionality reduction level, respectively;

Output: $offsetFile[]$ is an array of files used for saving the offset of the hashes to retrieve the reference IDs; $IDFile[]$ is an array of files used for saving the reference IDs of each hash.

Variables: $dictArr[][]$ and $subDictArr[][]$ consist of reference IDs for each hash value.

```
function getKssdIndexDict(S_a[M], half_K, drlevel, offsetFile[], IDFile[])
    if half_K − drlevel > 8 then
        numDict ← 2^{4×(half_K − drlevel − 8)};
        n ← 2^{32};
        for index = 0; index < numDict; index++ do
            subDictArr[n][];
            for i = 0; i < M; i++ do
                size ← S_a[i].size;
                for j = 0; j < size; j++ do
                    hash ← S_a[i][j];
                    curIndex ← hash/n;
                    curHash32 ← hash%n;
                    if curIndex == index then
                        subDictArr[curHash32].append(i);
                    end if
                end for
            end for
            saveDictFile32(subDictArr, offsetFile[index], IDFile[index]);
        end for
    else
        n ← 2^{4×(half_K − drlevel)};
        dictArr[n][];
        for i = 0; i < M; i++ do
            S ← S_a[i].size;
            for j = 0; j < S; j++ do
                hash32 ← S_a[i][j];
                dictArr[hash32].append(i);
            end for
        end for
        saveDictFile32(dictArr, offsetFile[0], IDFile[0]);
    end if
end function
```

Algorithm 6 Generating the *indexed dictionary* of RabbitKSSD

Input: Reference sketch array $S_a[M]$ contains M sketches; Each sketch ($S_a[i]$) is a hash array; $half_K$ and $drlevel$ are the parameters of k-mer size and dimensionality reduction level, respectively;

Output: $offsetFile$ is a file that contains the offset of each hash value for retrieving the reference IDs; $IDFile$ is a file that contains the reference IDs of each hash value;

Variables: $dictMap$ is a map of hash values and their reference IDs; $dictArr$ is a two-dimensional array of hash values and their reference IDs.

```
function getRabbitKSSDIndexDict(S_a[M], half_K, drlevel, offsetFile, IDFile)
    if half_K − drlevel > 8 then
        for i = 0; i < M; i + + do
            size ← S_a[i].size;
            for j = 0; j < size; j + + do
                hash64 ← S_a[i][j];
                dictMap[hash64].append(i);
            end for
        end for
        saveDictFile64(dictMap, offsetFile, IDFile);
    else
        n ← 2^{4×(half_K − drlevel)};
        dictArr[n][];
        for i = 0; i < M; i + + do
            S ← S_a[i].size;
            for j = 0; j < S; j + + do
                hash32 ← S_a[i][j];
                dictArr[hash32].append(i);
            end for
        end for
        saveDictFile32(dictArr, offsetFile, IDFile);
    end if
end function
```

Algorithm 7 Storing the *indexed dictionary* to files

Input: $dictArr$ is a two-dimensional array to store the hash values and reference IDs. $dictMap$ is the map data structure of hash and its reference IDs; For each element e in $dictMap$, $e.first$ is the hash value, $e.second$ is the array of reference IDs.

Output: $offsetFile$ is a file that contains the start retrieving offsets for each hash value; $IDFile$ is a file that contains the Reference IDs for each hash value.

Variables: $offsetArr[]$ contains offsets of each hash value to retrieve the reference IDs; $hashArr[]$ contains the hash value corresponds to the offset.

function saveDictFile32($dictArr, offsetFile, IDFile$)
    $n \leftarrow dictArr.size$; $offsetArr[n]$;
    for $i = 0; i < n; i++$ **do**
        $size \leftarrow dictArr[i].size$;
        if $i == 0$ **then**
            $offsetArr[i] \leftarrow size$;
        else
            $offsetArr[i] \leftarrow offsetArr[i-1] + size$;
        end if
        $WriteToFileAppend(IDFile, dictArr[i].data)$;
    end for
    $WriteToFile(offsetFile, offsetArr.data)$;
end function
function saveDictFile64($dictMap, offsetFile, IDFile$)
    $hashNum \leftarrow dictMap.size$;
    $hashArr[hashNum], offsetArr[hashNum]$;
    $i \leftarrow 0$;
    for e **in** $dictMap$ **do**
        $key \leftarrow e.first$;
        $hashArr[i] \leftarrow key$;
        $size \leftarrow e.second.size$;
        if $i == 0$ **then**
            $offsetArr[i] \leftarrow size$;
        else
            $offsetArr[i] \leftarrow offsetArr[i-1] + size$;
        end if
        $WriteToFileAppend(IDFile, e.second.data)$;
        $i \leftarrow i + 1$;
    end for
    $WriteToFileAppend(IDFile, hashArr.data)$;
    $WriteToFile(offsetFile, offsetArr.data)$;
end function
